# Dynamic modeling of geological carbon storage in an oil reservoir, Bredasdorp Basin, South Africa

**DOI:** 10.1038/s41598-023-43773-9

**Published:** 2023-10-03

**Authors:** Blessing Afolayan, Eric Mackay, Mimonitu Opuwari

**Affiliations:** 1 Petroleum Geosciences Research Group, Department of Earth Sciences, University of the Western Cape, Bellville, 7535 Republic of South Africa; 2 Institute of Petroleum Engineering, Heriot-Watt University, Riccarton, Edinburgh, EH14 4AS UK

**Keywords:** Climate sciences, Environmental sciences

## Abstract

Geological carbon storage provides an efficient technology for the large-scale reduction of atmospheric carbon, and the drive for net-zero emissions may necessitate the future usage of oil reservoirs for CO_2_ projects (without oil production), hence, dynamic modeling of an oil reservoir for CO_2_ storage in the Bredasdorp basin, South Africa, was therefore conducted. Injection into the reservoir was for 20 years (2030–2050), and 100 years (2050–2150) to study the CO_2_–brine–oil interactions, with sensitivities carried out on reservoir boundary conditions. The closed boundary scenario experienced pressure buildup with a target injection rate of 0.5 Mt/year, and a cutback on injection rate progressively until 2050 to not exceed the fracture pressure of the reservoir. The CO_2_ plume migration was not rapid due to the reduced volume of CO_2_ injected and the confining pressure. The system was gravity dominated, and gravity stability was not attained at the end of the simulation as fluid interfaces were not yet flat. The open boundary reservoir did not experience a pressure buildup because all boundaries were open, the target injection rate was achieved, and it was a viscous-dominated system. In both cases, the dissolution of CO_2_ in oil and brine was active, and there was a growing increase of CO_2_ fraction dissolved in water and oil, a decline in gaseous mobile CO_2_ phase between 2050 and 2150, and active trapping mechanisms were structural trapping, dissolution in oil and water, and residual trapping. The study showed that boundary condition was very crucial to the success of the project, with direct impacts on injection rate and pressure. This pioneering study has opened a vista on the injection of CO_2_ into an oil reservoir_,_ and CO_2_–brine–oil interactions, with sensitivities carried out on reservoir boundary conditions in a closed and an open hydrocarbon system in South Africa.

## Introduction

Global sea level and temperature rise (climate change) have been constantly perceived to be caused by the untoward concentration of greenhouse gases (GHGs) in the earth’s atmosphere, and carbon dioxide (CO_2_) the major constituent of GHGs^1–6^. Other energy sources especially renewables have received significant investments, but due to the ever-increasing demand for energy globally, fossil fuels will still be in the mix for the foreseeable future^1,7,8^. With man’s continued reliance on fossil fuels, and CO_2_ being a byproduct of fossil fuel combustion, drives and initiatives to mitigate the adverse effects of climate change must constantly be on the front burner. Goal 13 (climate action), one of the goals within the Sustainable Development Goals (SDGs) as adopted in 2015 by the United Nations (UN) speaks to the necessity of taking “urgent actions to combat climate change and its impact” and targets the integration of climate change strategies into national planning and policies^9,10^. Geological carbon storage is presently the most efficient technology for large-scale reduction of atmospheric carbon and stemming the upward tide of associated climate challenges, as the International Energy Agency asserts it can bring about around 19% reduction in emissions of CO_2_ worldwide by 2050^11^. Yearly CO_2_ emissions on a global scale from cement manufacture and burning of fossil fuels were 36.58 Giga tonnes per annum (Gtpa) in 2018, with 10.06 Gt (28% of the world’s entirety) contributed by China, the USA emitted 5.42 Gt (15% of the world’s total sum), and India accounted for 2.65 Gt, representing 7% of total CO_2_ emission of the world^12,13^. With 28 carbon capture and storage (CCS) projects operational worldwide as of 2020 and sequestering 41 Mtpa^14^, this represents a meagre 0.12% of the CO_2_ emissions attributable to hydrocarbon and cement industries worldwide. About 37% of these are principally geological carbon storage projects, while 73% are for enhanced oil recovery (EOR) drives, with the USA, Brazil and, Australia being the nations that sequestered the most^13^. Some operational carbon storage projects around the world include the Gorgon Carbon Dioxide Project, Uthmaniyah CO_2_-EOR project^15–17^, CNPC Jilin Oil Field CO_2_-EOR^18–20^ and Alberta Carbon Trunk Line (ACTL) Sturgeon Refinery CO_2_ Stream project^21–23^. Some under construction presently include The Sinopec Qilu Petrochemical Carbon Store Project^24,25^, Guodian Taizhou Power Station Carbon Capture^26,27^, Air Liquide Refinery Rotterdam CCS^28–30^, The ZEROS Project^31,32^ and Norcem Brevik Cement Plant^33,34^ among others.

**Figure 1:**
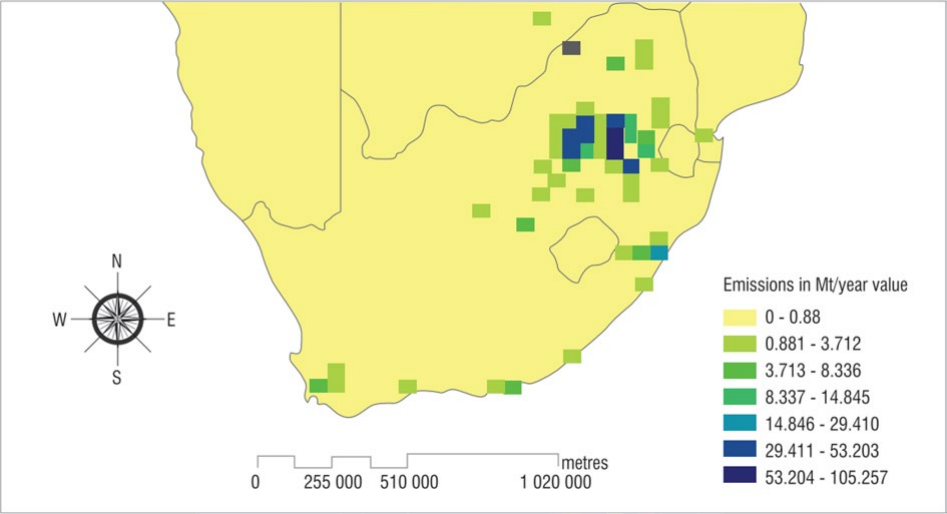
Core point sources of carbon dioxide emissions in South Africa are in the Free State, Gauteng, and Mpumalanga provinces hosting the coal mines and most of the coal-fired power plants. Emissions from oil refineries, gas-to-liquid, and coal-to-liquid firms are also covered here. Adapted after^48^.

Within the South African context, studies have shown that South Africa has the potential of storing ~ 150 Gt of CO_2_, with the offshore basins showing greater prospects^35–40^, but it is unlikely that commercially available CCS will be in place in the country before 2030 due to the present challenges of doubts in policies around climate action, delayed pilot/test underground carbon injection projects, low-priced carbon rates, dearth of commercial or profitable case studies, and the acceptability of the public to carbon storage projects in regions hosting potential storage sites^41–47^. South Africa fulfils its energy needs principally from fossil fuels, with 77% of the nation’s total electricity generation from coal-fired power plants and its power sector the 9th biggest emitter among power sectors in the world (~ 218 Mt of CO_2_) (Fig. 1), though these are low emissions in comparison to developed nations, cumulative emissions from the next nine African nations are below it^48^. Preliminary studies have been carried out to identify and estimate the capacities of potential geological CO_2_ reservoirs in South Africa^39^, with particular focus on the Zululand basin^49^, Algoa^36,50^, and the Bredasdorp basin^51–53^, with a pilot CO_2_ injection test scheduled for 2023^54,55^. These continued inquests into underground carbon storage are a pointer that it will play a big part in the decarbonization of South Africa.

In the bid to attain climate neutrality, Denmark, currently the largest oil producer in the European Union (EU), is set to completely phase out the production of hydrocarbon, new oil and gas extraction permits have been cancelled and when the calendar reads 2050, the hydrocarbon valves will be shut off for good. Presently, subsidies are being provided to stimulate large-scale CCS projects^56,57^. This means more searchlights will be beamed on oil reservoirs (without oil recovery) to make them viable CO_2_ sinks. Oil reservoirs present many advantages for CO_2_ storage, such as the presence of below-surface and on-the-ground installations and equipment that can be tailored to CO_2_ injection and storage (usually with some modifications), the presence of quality seal and establishment of caprock integrity which have held oil in place through geologic time, and availability of geological, hydrogeological, geophysical and engineering data for characterization of the reservoir and other petroleum system elements, among others^5,58–64^.

Therefore, this research aims to consider an oil reservoir in the offshore Bredasdorp basin, South Africa, for CO_2_ storage, with no enhanced oil recovery (EOR). The knowledge gained from the study will also be useful for reservoirs with pockets of unrecoverable oil due to economic, technical, commercial, or logistics reasons, and bypassed oil/unswept zones.

## Methodology

With the aid of the equation-of-state CMG-GEM compositional simulator (2022.30v), dynamic simulation was conducted for CO_2_ injectivity and storage in the oil reservoir, with a focus on CO_2_ plume migration, active trapping mechanisms, and supercritical CO_2_–oil–brine interactions. The reservoir used for this study is a clastic type with shales, siltstones, and sandstone units, and it has a permeability range of 3–560 mD and average effective porosity of 14%^51^. For CO_2_ solubility in the aqueous phase, Henry’s law was employed^65–69^, with the formation and fluid values, as well as the composition of the oil used for the study presented in Tables 1, 2.

**Table 1:** Reservoir and fluid values.

Reservoir and fluid properties	Values
Type of reservoir	Sandstone
Maximum thickness (m)	77
Depth of reservoir top (m)	2622
Pressure at reservoir depth (kPa)	26,153
Temperature at reservoir depth (°C)	110
Rock compressibility (kPa)	5.29 × 10^−7^
Maximum bottom hole pressure (kPa)	30,000
Number of injection wells	1
Model discretization	89 × 72 × 40
Total blocks	256,320
Number of active blocks	240,892
Water density (kg/m^3^)	1010
Rate of injection (m^3^/day)	762,000
Irreducible water saturation (rock type 1)	0.144
Irreducible water saturation (rock type 2)	0.294
Residual oil saturation (rock type 1)	0.423
Residual oil saturation (rock type 2)	0.326
Permeability cut-off for rock type (mD)	150

**Table 2:** Oil composition.

Composition	Molar fraction
CH_4_	21.33
C_2_H_6_	7.39
C_3_H_8_	6.05
C_4_H_10_	2.41
C_5_H_12_	3.44
C_6_H_14_	3.37
C_7_H_16_–C_13_H_28_	30.18
C_14_H_30_–C_20_H_42_	10.7
C_21_H_44_–C_28_H_58_	5.55
C_29_H_60_ +	9.57

**Figure 2:**
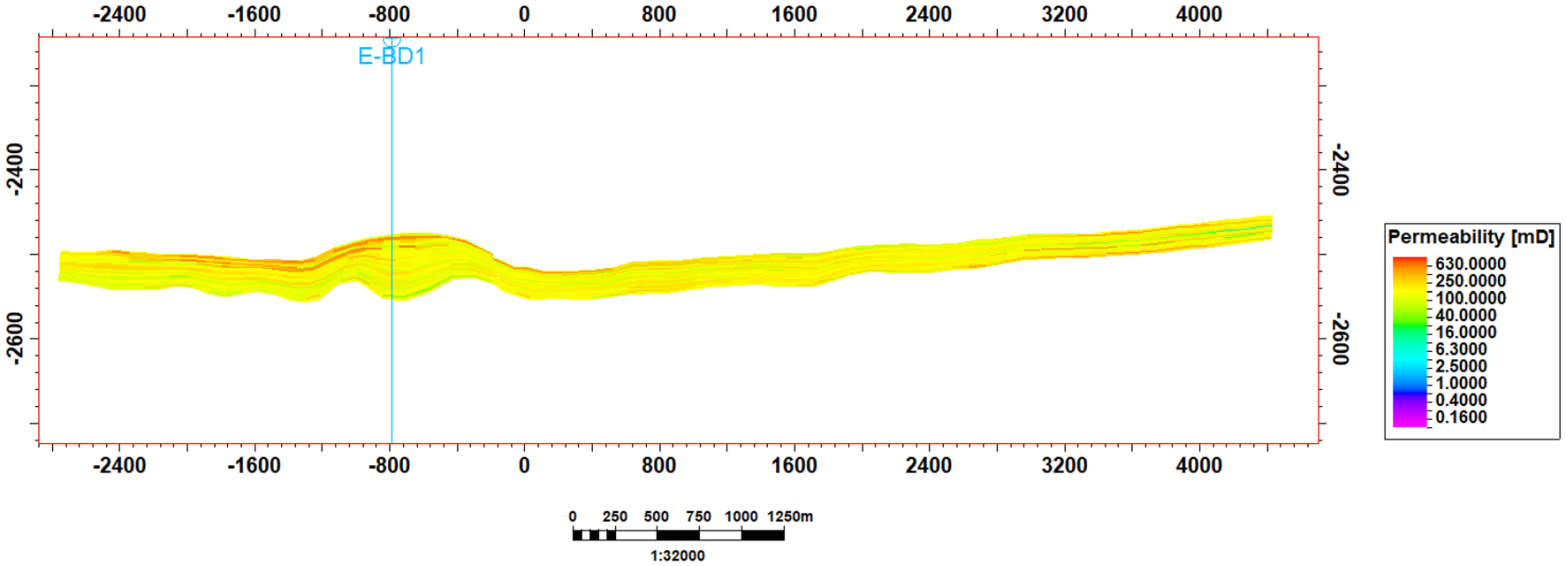
EW cross-sectional view of permeability distribution in the reservoir.

**Figure 3:**
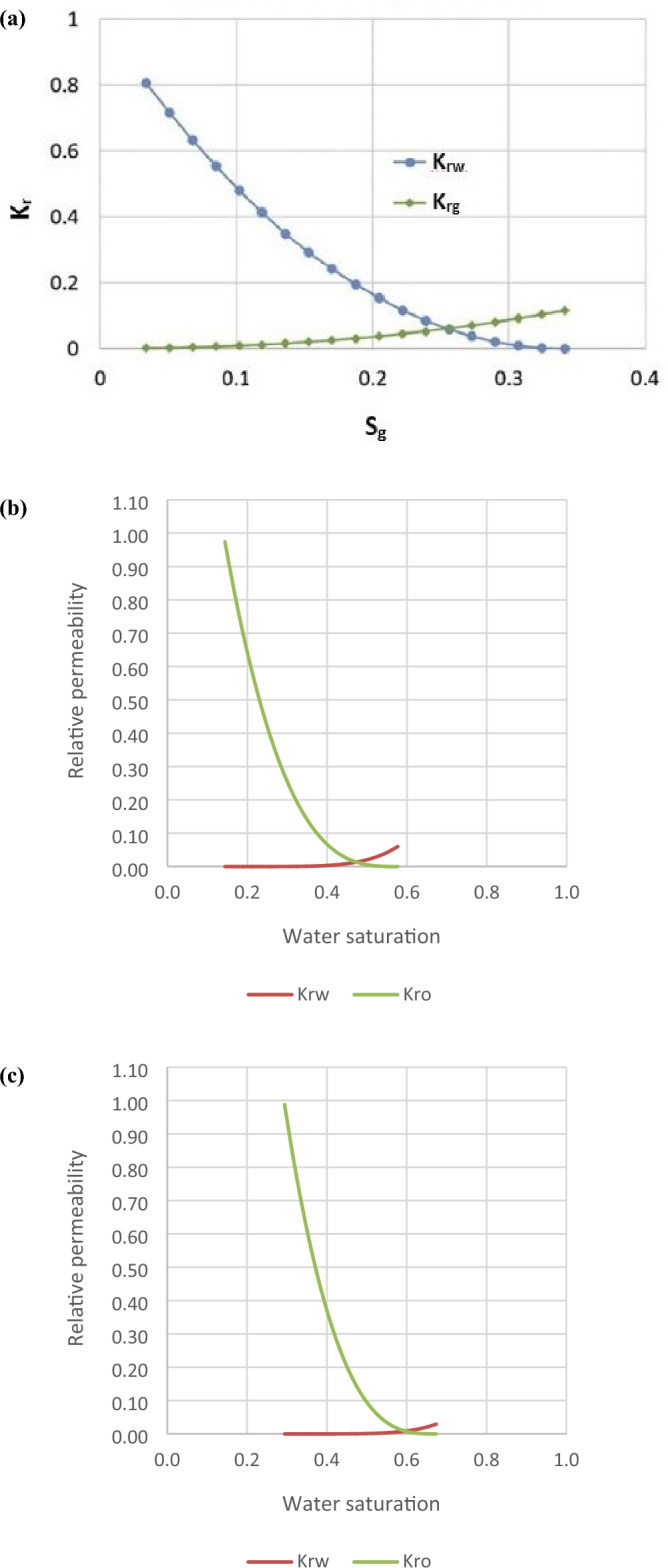
(**a**) Liquid–gas relative permeability curve; (**b**) water–oil relative permeability curve for high permeability zones and (**c**) water–oil relative permeability curve for low permeability zones.

Two rock types were delineated in the reservoir based on permeability zones^70^, namely high (rock type 1) and low (rock type 2) permeability zones, with permeability cut-off set at 150 mD as the reservoir had prevailing permeability values of 100–560 mD^51^ (Fig. 2). Given three-phase flow in the reservoir (oil, CO_2_, and water) and at connate water saturation, for both zones, there were liquid–gas relative permeability values^71^, and water–oil relative permeability values (generated from Corey correlation)^72–76^ (Fig. 3).

Dynamic simulations were carried out to simulate the injection of 0.5 Mt/year of CO_2_ (762,000 m^3^/day) in the oil reservoir for 20 years (2030–2050) with one injection well, and a further simulation period of 100 years (2050–2150) to study the CO_2_–oil–brine interactions. Two scenarios were considered based on the boundary conditions of the reservoir, namely (i) open system (all boundaries open); and (ii) closed system (all boundaries closed).

## Results and discussion

### Closed boundary system

#### Injection and pressure

**Figure 4:**
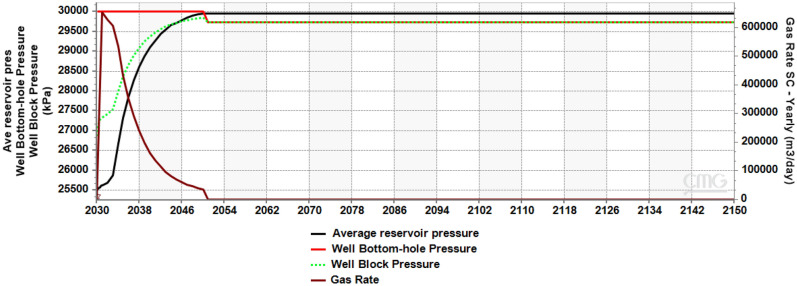
Cross-plot of average reservoir pressure, well bottom-hole pressure, well block pressure and gas rate.

The maximum allowable pressure (well bottom-hole pressure, BHP) in the reservoir was set at 30,000 kPa, equivalent to 90% of reservoir lithostatic pressure^77,78^. With the target gas rate set at 0.5 Mt/year (762,000 m^3^/day) and the inability of pressure to dissipate in the system due to closed reservoir boundaries, the plot of BHP against time (Fig. 4) showed a steadily rising gas rate until it reached the maximum rate allowable (653,649 m^3^/day) to not exceed the maximum pressure, then the injection/gas rate was cut back to ensure caprock was not damaged. The average reservoir pressure rose from 25,500 kPa at the onset of injection in 2030, peaked at 30,000 kPa in 2050 and this pressure was maintained from 2050 to 2150. BHP and Well block pressure dropped to 29,741 kPa in 2050 to 2150, as the pressure due to injection of CO_2_ into the reservoir had ceased.

In any underground carbon storage project, a target amount of CO_2_ is proposed for injection into the ground, usually accompanied by pressure buildup in the system. The attainment of this desired injection rate is dependent on the injectivity of the reservoir, as an injection rate that is too high can cause the downhole pressure to exceed the fracture pressure, inducing reservoir and seal fracturing, or reactivation of existing sealing faults, all leading to environmental concerns^64^. A typical project that has had to deal with this scenario is the CO_2_ injection into the Tubåen Formation at Snøhvit, where a considerable rate of pressure at the early onset of the project led to cutting down of injection rates, and subsequent abandonment of injection into the formation when the pressure increase considerably approached the fracture pressure^79–81^.

#### CO_2_ plume migration and active flow regime

**Figure 5:**
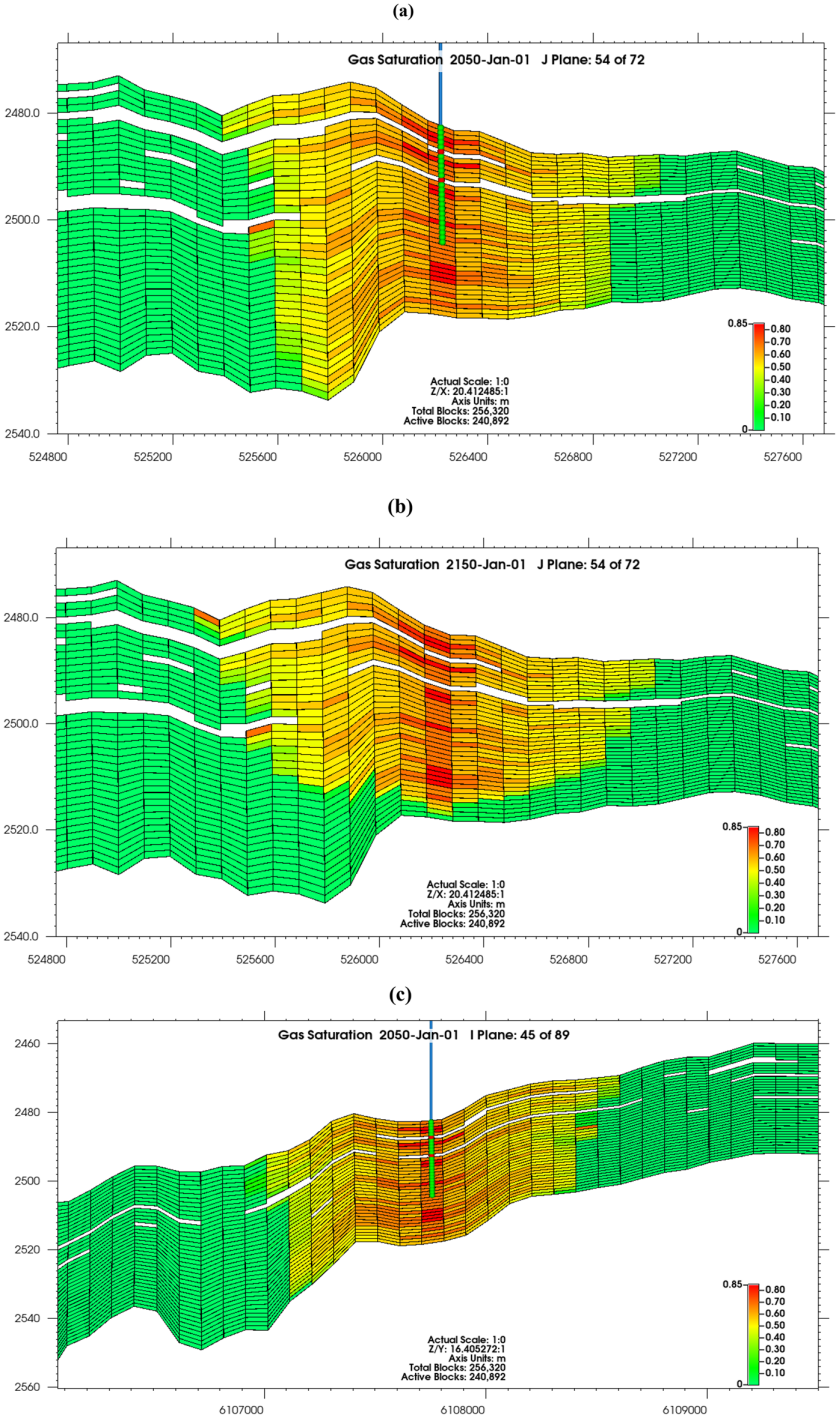

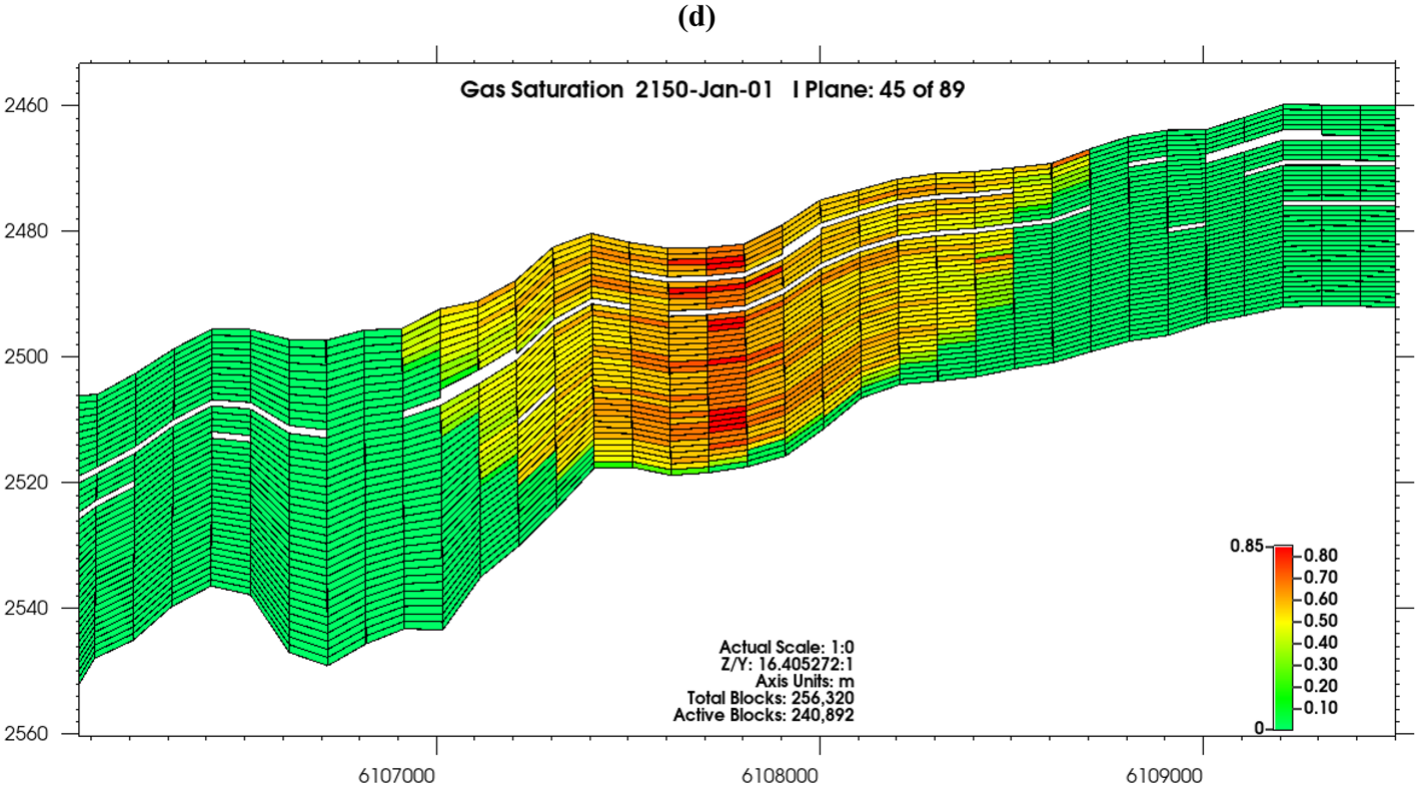
Gas saturation profile from (**a**) J plane after 20 years of injection (2050), (**b**) J plane at 100 years of storage (2150), (**c**) I plane after 20 years of injection (2050), (**d**) I plane at 100 years of storage (2150).

CO_2_ plume did not migrate rapidly away from the wellbore due to confining pressure, and reduction in the volume of CO_2_ injected into the reservoir with the inability to attain the intended constant injection rate of 0.5 Mt/year (Fig. 5a,c). Supercritical CO_2_ accumulated at the top of the reservoir due to buoyancy in 2150, rather than moving rapidly laterally (Fig. 5b,d)^82^. The system was gravity dominated, as counter-current movement ensued in the vertical route owing to the gravity segregation of CO_2_, water, and oil, and the CO_2_ invasion into the reservoir being stable due to gravity stability and gravity exceeding viscous forces^83^, it is also pertinent to note that low injection rate is accompanied with less lateral spreading of gas^84^. Though the gravity segregation was reduced because of relative permeability^85–88^, with increasing residence time, the system will be fully gravity stable evidenced by fluid interfaces being flat, as interfaces are usually destabilized by viscous forces^83^. There was structural trapping under the caprock, and the occurrence of non-reservoir rocks also provided local compartmentalization in the reservoir, aiding the reduction of the rapid migration of the plume.

#### CO_2_ dissolution in oil and brine

**Figure 6:**
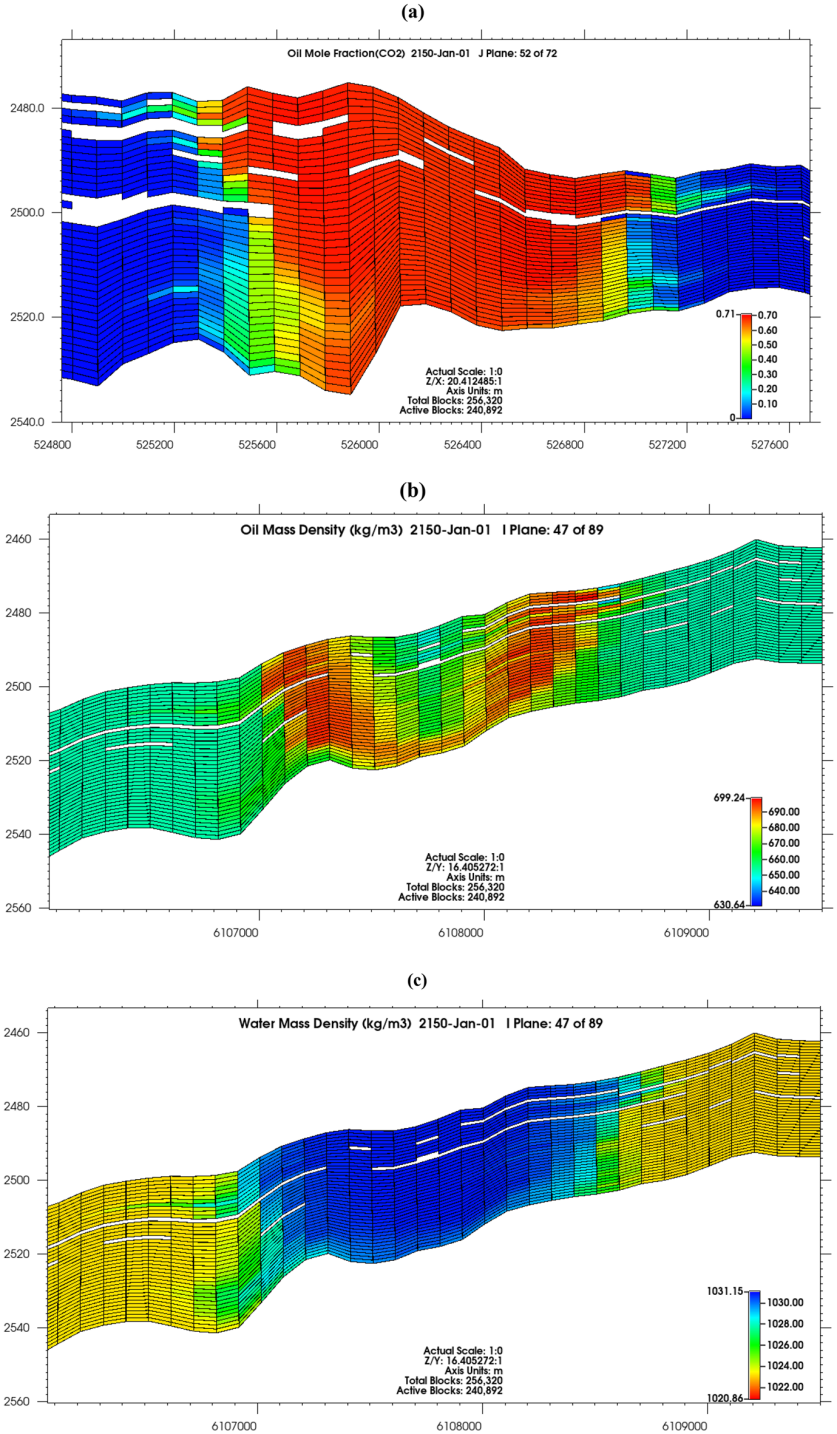
(**a**) Oil mole fraction of CO_2_ in 2150; (**b**) Oil mass density in 2150; (**c**) Water mass density in 2150.

Displacement of oil by injected CO_2_ is active in the reservoir, though only 71% of the oil can be displaced (Fig. 6a), the heavier hydrocarbon components (29%) are not dissolved into the gas phase. Oil mass density (Fig. 6b) ranged from 650 kg/m^3^ in the CO_2_ uninvaded zones to 699 kg/m^3^ in the zones invaded by CO_2_, with water mass density (Fig. 6c) also increasing to 1031 kg/m^3^ in blocks that CO_2_ had invaded. This indicates active dissolution of CO_2_ in the reservoir fluids, as formation fluids density increases progressively as they become enriched with CO_2_^89,90^.

#### Active trapping mechanism

**Figure 7:**
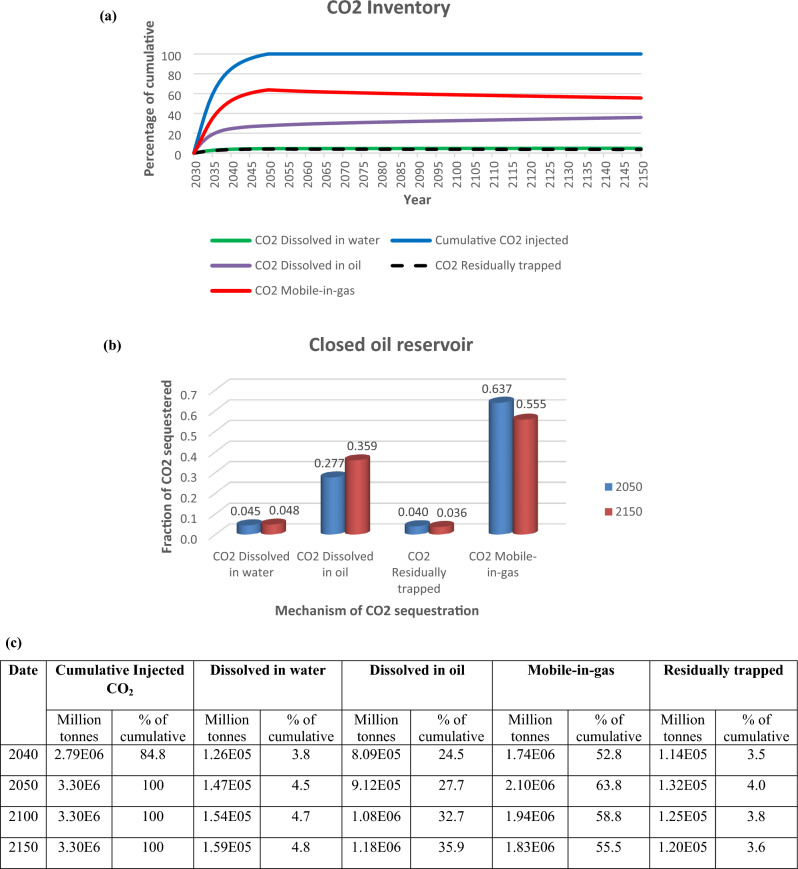
(**a**) CO_2_ trapping mechanism, (**b**) fraction of CO_2_ sequestered, (**c**) summary of volumes of CO_2_ injected, and in different trapping mechanisms.

Active CO_2_ trapping mechanisms were structural trapping under the caprock (supercritical mobile), dissolution and residual trapping (Fig. 7a,c). At the end of injection in 2050, 74.9 billion moles (3.3 Mt) of CO_2_ had been successfully injected into the reservoir, 63.7% was in the gaseous mobile phase, 27.7% had dissolved in oil, 4% was trapped residually and 4.5% had dissolved in brine (Fig. 7b,c). In 2150, 4.8% had dissolved in water, 35.9% dissolved in oil, 3.6% was trapped residually and 55.5% was in the gaseous mobile phase.

There was a growing increase in CO_2_ fraction dissolved in oil and water and a decline in the residually trapped and gaseous mobile CO_2_ phase. With further residence time, which is a core element of the CO_2_ storage process^91–93^, this trend should continue.

### Open boundary system

#### Injection and pressure

**Figure 8:**
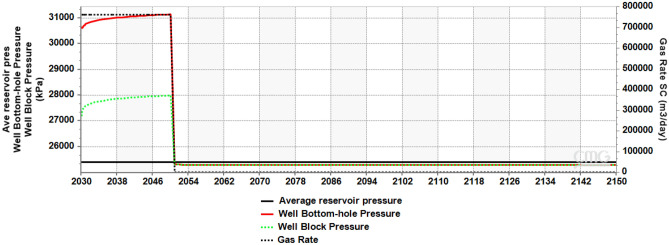
Cross-plot of gas rate, average reservoir pressure, well block pressure and well bottom-hole pressure.

With all boundaries open, there was pressure dissipation in the system. In Fig. 8, the average reservoir pressure was constant at 25,395 kPa from 2030 to 2150, maximum well bottom-hole pressure recorded was 31,000 kPa in 2050, which was below the lithostatic pressure of the formation (33,440 kPa). The pressure dropped to 25,290 kPa after cessation of injection, which was maintained till 2150. Well block pressure rose from 26,000 kPa in 2030 to 26,297 kPa in 2050 and dropped to 25,290 kPa from 2050 to 2150. Pressure dissipation in the system because of open boundaries also ensured the proposed gas injection rate of 762,000 m^3^/day (0.5 Mt/year) was fully attained, with no direct risks of caprock and reservoir fracture, or storage capacity destruction of the rock^94^.

#### CO_2_ plume migration and active flow regime

**Figure 9:**
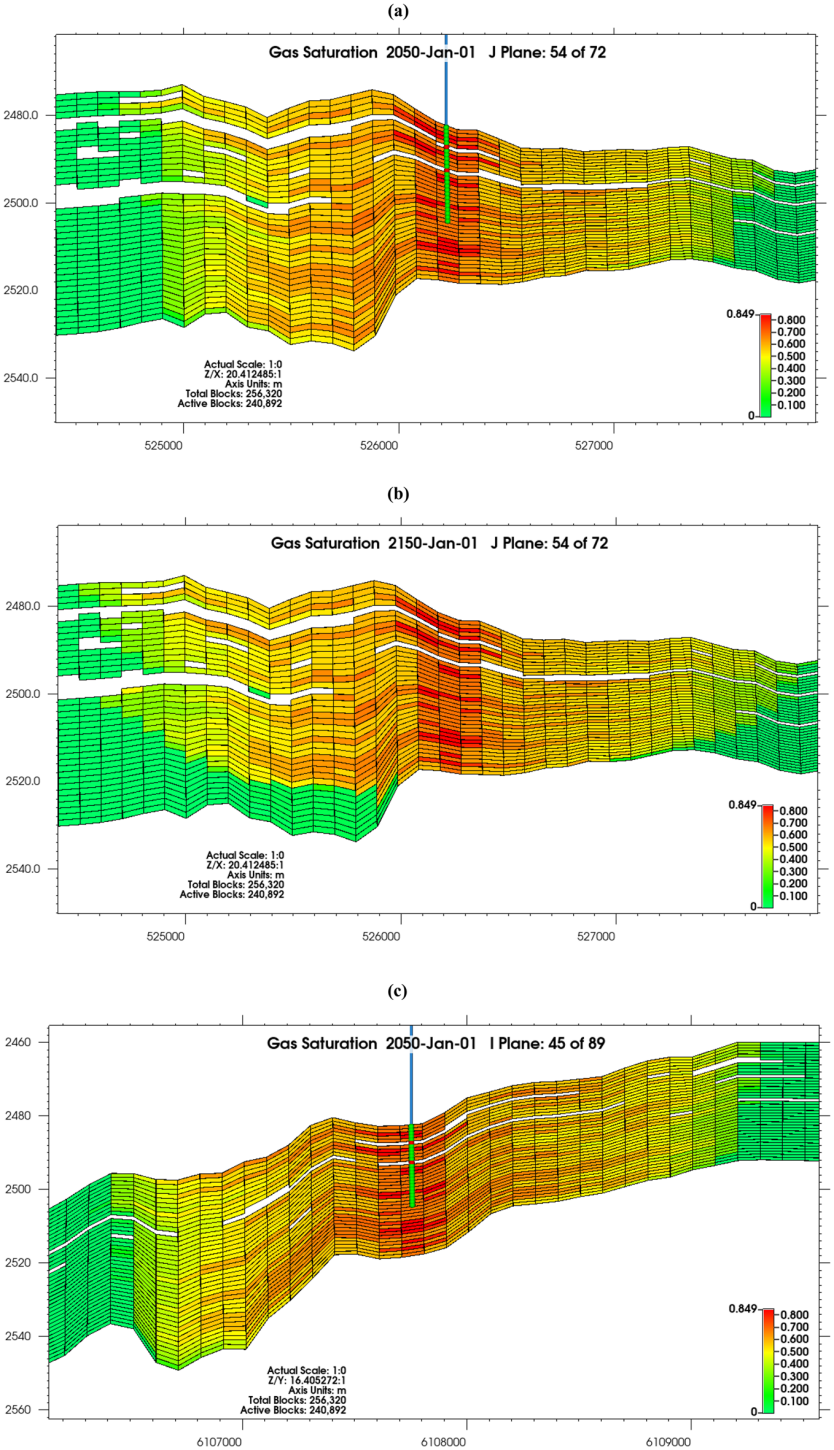

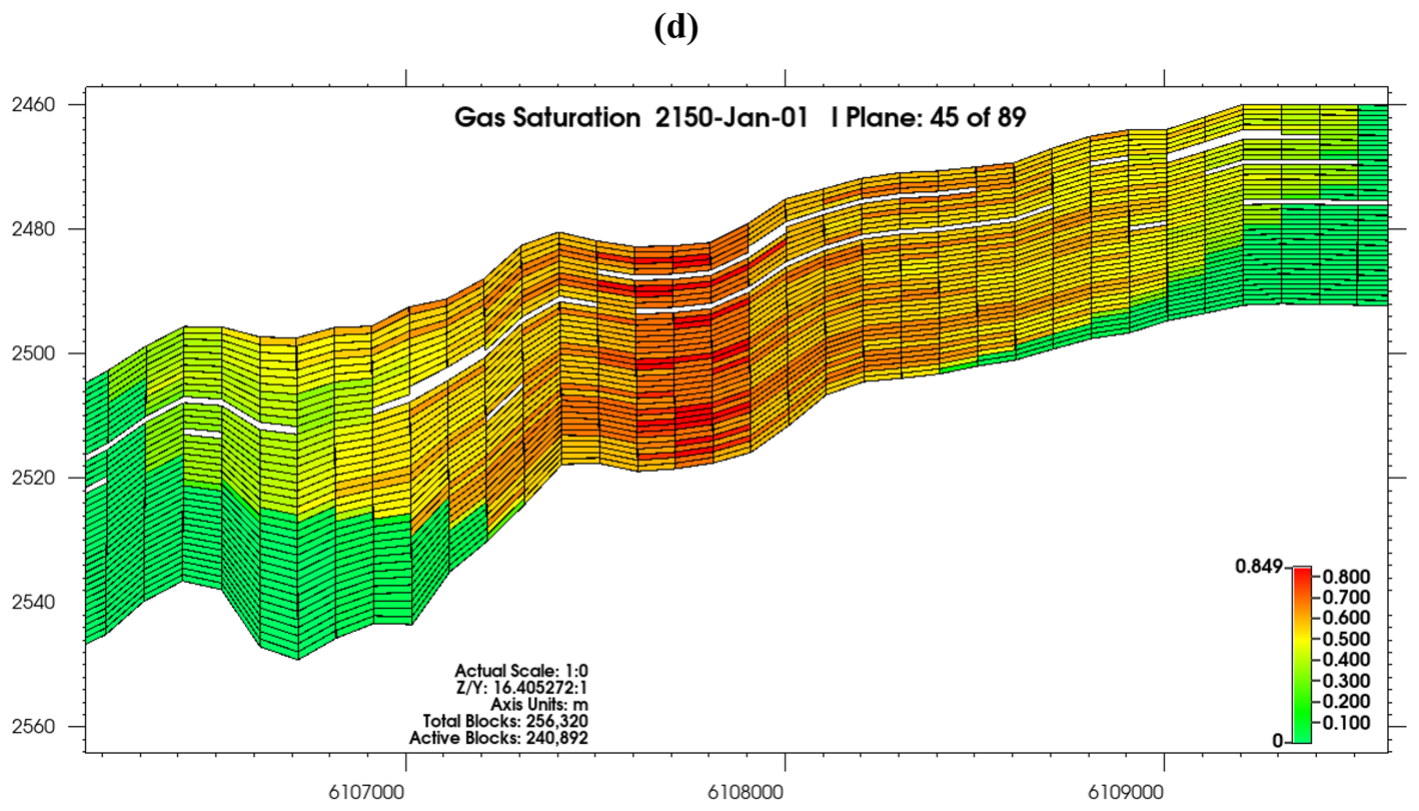
(**a**) Gas saturation shown from the J plane after 20 years (at 2050), (**b**) gas saturation in 2150 shown from the J plane, (**c**) gas saturation shown from the I plane after 20 years (at 2050), and (**d**) gas saturation in 2150 shown from the I plane.

There was gradual and active lateral migration of injected CO_2_ away from the injection well (Fig. 9a–d), as there was no confining pressure in the reservoir. The system was viscous-dominated as increased injection rates were accompanied by increasing viscous forces^95^, though gravity was also active, as shown by up-dip movement (Fig. 9c,d) and vertical segregation of the CO_2_ plume from the bottom of the reservoir due to buoyancy, with supercritical CO_2_ accumulating and moving directly below the seal but did not get to the reservoir flanks (Fig. 9b,d).

#### CO_2_ dissolution in oil and brine

**Figure 10:**
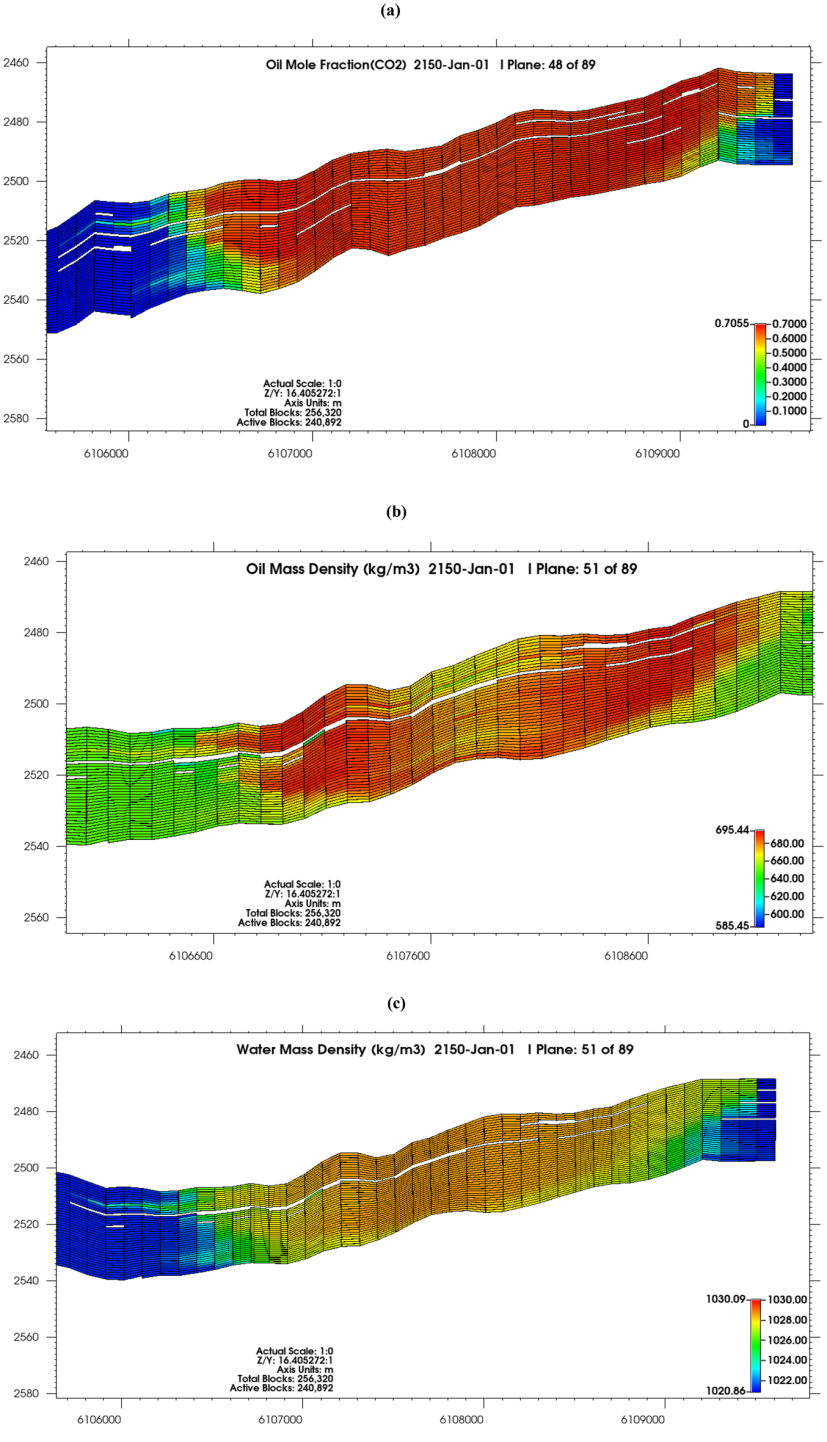
(**a**) Oil mole fraction of CO_2_ in 2150; (**b**) oil mass density in 2150; (**c**) water mass density in 2150.

Oil displacement by injected CO_2_ was active in the reservoir, though only 70.5% of the oil could be displaced (Fig. 10a), the heavier hydrocarbon components (29.5%) are not dissolved into the gas phase. Oil mass density (Fig. 10b) ranged from < 640 kg/m^3^ in the CO_2_ uninvaded zones to 695 kg/m^3^ in the zones invaded by CO_2_, with water mass density (Fig. 10c) also increasing from 1021 kg/m^3^ in uninvaded zones to 1030 kg/m^3^ in blocks that CO_2_ had invaded. This is indicative of the active dissolution of CO_2_ in the reservoir fluids, as formation fluids density increases progressively as they become CO_2_ enriched^89,90^.

#### Active trapping mechanism

**Figure 11:**
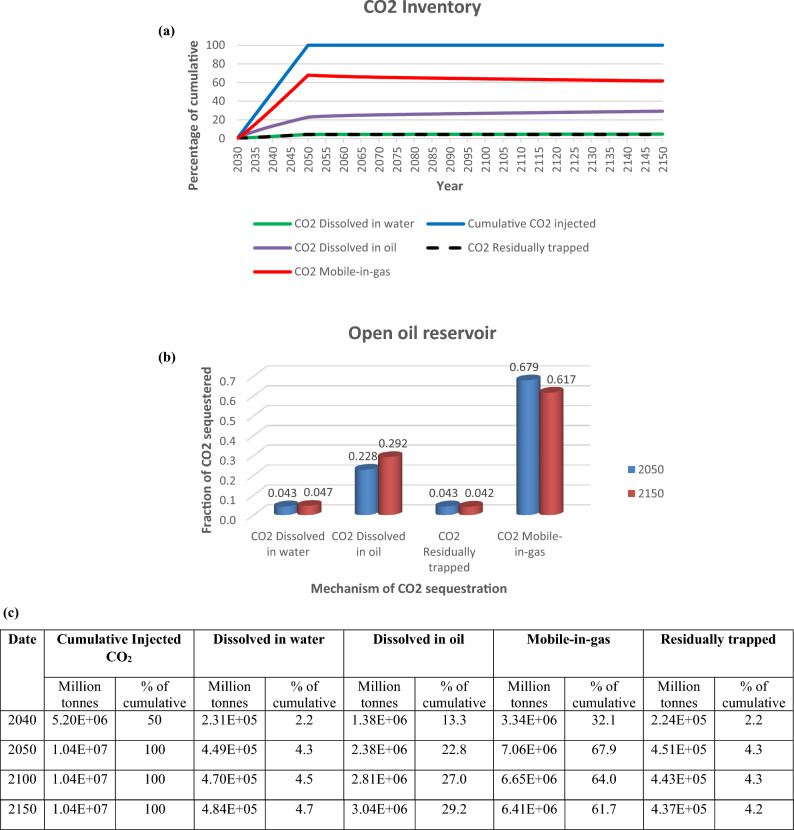
(**a**) CO_2_ trapping mechanism, (**b**) fraction of CO_2_ sequestered, (**c**) summary of volumes of CO_2_ injected, and in different trapping mechanisms

Active CO_2_ trapping mechanisms were structural trapping under the caprock (supercritical mobile), dissolution in oil and brine, and residual trapping (Fig. 11a,c). At the end of injection in 2050, 236 billion moles (10.4 Mt) of CO_2_ had been successfully injected into the reservoir, 67.9% was in the gaseous mobile phase, 22.8% had dissolved in oil, 4.3% was trapped residually and 4.3% had dissolved in brine (Fig. 11b,c). In 2150, 4.7% had dissolved in water, 29.2% dissolved in oil, 4.2% was trapped residually and 61.7% was in the gaseous mobile phase.

There was a gradual decline in the gaseous mobile CO_2_ phase and an increase in the CO_2_ fraction dissolved in oil and water. This leaning should continue upon further residence time, and a marked increase in CO_2_ dissolution in water occasioned by an influx of brine into the reservoir.

## Conclusion

In this study, an oil reservoir in the offshore lying Bredasdorp basin, South Africa, has been considered for CO_2_ storage, without enhanced oil recovery (EOR). The closed boundary scenario experienced a pressure buildup with a target injection rate of 0.5 Mt/year, and therefore a cutback on injection rate progressively until 2050 to ensure the reservoir and overlying seal were not damaged. Migration of the CO_2_ plume was not rapid, due to the reduced volume of CO_2_ that was injected and confining state of the reservoir, the system was gravity dominated but did not attain gravity stability at the end of the simulation. There was a growing increase of CO_2_ fraction dissolved in water and oil and a decline in the gaseous mobile CO_2_ phase between 2050 and 2150. In 2150, 4.8% had dissolved in water, 35.9% dissolved in oil, 3.6% was trapped residually and 55.5% was in the gaseous mobile phase. The open boundary state experienced no pressure buildup in the reservoir and the target injection rate of 0.5 Mt/year was achieved, and 10.4 Mt of CO_2_ had been successfully injected into the reservoir. CO_2_ plume migrated up-dip without getting to the reservoir flanks, it was a viscous-dominated system attended with gravity movement and segregation. With an increase in the density of formation fluids, the dissolution of CO_2_ in brine and oil was active, active trapping mechanisms were structural trapping, dissolution in oil and water and residual trapping. There was a decline in the gaseous mobile CO_2_ phase and an increase in CO_2_ fraction dissolved in oil and water between 2050 and 2150.

With further residence time, fractions of CO_2_ dissolved in the oil and brine phases would increase, as well as residually trapped fractions, with the CO_2_ gaseous mobile phase experiencing a continuous decline. Therefore, this study showed that boundary condition was key to the success of the project, as it impacts injection rate and pressure.

## Data Availability

The data that support the findings of this study are available from the Petroleum Agency of South Africa (PASA) but restrictions apply to the availability of these data, which were used under license for the current study, and so are not publicly available. Data are however available from the authors upon reasonable request and with permission of the Petroleum Agency of South Africa (PASA).
